# A longitudinal study on diarrhoea and vomiting in young dogs of four large breeds

**DOI:** 10.1186/1751-0147-54-8

**Published:** 2012-02-02

**Authors:** Bente K Sævik, Ellen M Skancke, Cathrine Trangerud

**Affiliations:** 1 Department of Companion Animal Clinical Sciences, Norwegian School of Veterinary Science, P.O.Box 8146, Dep., N-0033 Oslo, Norway

**Keywords:** longitudinal study, diarrhoea, vomiting, incidence, risk factors, dog

## Abstract

**Background:**

Prospective studies to document the occurrence of canine diarrhoea and vomiting are relatively scarce in dogs, and the majority of published studies are based on information from clinical records. This study investigates the incidence risk of diarrhoea and vomiting as well as potential risk factors.

**Methods:**

A cohort study of 585 privately owned dogs of four breeds: Newfoundland, Labrador retriever, Leonberger, and Irish wolfhound. The owners maintained a continuous log regarding housing, exercise, nutrition, and health of their dogs. Episodes of diarrhoea and vomiting were recorded in a consecutive manner in a booklet. The owners completed the questionnaires and reported information at three, four, six, 12, 18, and 24/25 months of age, called observational ages.

Associations with potential risk factors for diarrhoea and vomiting were investigated in separate generalized estimating equation analyses.

**Results:**

The incidence of both diarrhoea and vomiting was influenced by breed. Both diarrhoea and vomiting were relatively common in young dogs, occurring most frequently during the first months of life. After three months of age, the odds of diarrhoea were significantly lower when compared to the observational period seven weeks to three months (OR ranging from 0.31 to 0.70 depending on the period). More males than females suffered from diarrhoea (OR = 1.42). The occurrence of diarrhoea was more common in dogs that also experienced episode(s) of vomiting during the study period (OR = 5.43) and *vice versa *(OR = 5.50). In the majority of dogs episodes of diarrhoea and vomiting did not occur at the same time. Dogs in urban areas had higher odds (OR = 1.88) of getting diarrhoea compared to dogs living in rural areas. The occurrence of both diarrhoea and vomiting demonstrated a seasonal variation with higher incidence during the summer months.

**Conclusion:**

Both diarrhoea and vomiting occurred most frequently during the first months of life. The incidence of diarrhoea and vomiting was significantly different between breeds. Diarrhoea occurred more frequently in males and in dogs living in urban areas. Also, a positive association between the occurrence of diarrhoea and vomiting in the same dog was found.

## Background

Prospective studies to document the occurrence of diarrhoea and vomiting are relatively scarce in dogs, and the majority of published studies are based on information from clinical records in veterinary hospitals. However, information from such databases might not be representative for the general population of dogs [1]. Diarrhoea and vomiting often occur as self-limiting episodes with few concerns for the owner, and no need for a veterinary consultation [2,3]. In such situations the owners might be more prone to "wait and see", compared to situations where the clinical signs are not so familiar. Thus, studies estimating occurrence of diarrhoea and vomiting based on clinical records will underestimate their true occurrence.

The reported occurrence of diarrhoea and vomiting in dogs is diverging between different studies. In a study of dogs purchased from an animal shelter in Northern Ireland, the prevalence of diarrhoea and vomiting within two weeks of acquisition was calculated to be 35.1% and 21.1%, respectively [4]. In another, more recent study, dog owners in Great Britain reported the occurrence of diarrhoea and vomiting during a two week period to be 14.9% and 18.9%, respectively [3]. However, a much lower frequency was reported for both diarrhoea and vomiting in two other studies. Edwards *et al. *[2] reported a frequency of 2.2% for both diarrhoea and vomiting in a period of two weeks prior to the survey. In a larger study from private veterinary practices in the United States the reported frequency of diarrhoea was 2.2% and for vomiting 2.1% during a one year period [5].

The lack of concurrence regarding the occurrence of diarrhoea and vomiting in canine populations might reflect differences in both study population characteristics and study-designs. Most published studies lack information on potential differences among breeds and gender. To the authors' knowledge there are no studies evaluating differences between urban and rural areas.

Hence, information from prospective studies regarding the incidence of diarrhoea and vomiting in a canine population and furthermore, how the occurrence is affected by potential risk factors are scarce, thereby initiating the presentation of results from this longitudinal study. The aim of the present study was to report owner recorded episodes of diarrhoea and vomiting, and potential risk factors for their occurrence. The hypothesis was that signalment and living location influenced the odds of diarrhoea and vomiting.

## Methods

The study was carried out in agreement with the provisions enforced by the Norwegian National Animal Research Authority.

### Study design

The present study is a part of a cohort study (the main study) conducted as a prospective field study, to investigate skeletal diseases in four large breeds: Newfoundland (NF), Labrador retriever (LR), Leonberger (LEO), and Irish wolfhound (IW) [6-12]. Seven hundred privately-owned dogs from 106 different litters from a total of 98 dams, belonging to 79 breeders, were included, each with a housing and feeding regimen decided by its owner.

### Inclusion of dogs

Inclusion of a litter in the project started when the bitch was mated. Each dog breeder, dog owner, and veterinarian participating in the project signed a written agreement of cooperation to comply with the project plan. All puppies were registered in the Norwegian Kennel Club. Not all dogs initially enrolled in the study, continued to completion. Reasons for non-completion included, but not limited to: death of the dog and relocation of the owners during the study. Additionally, for unknown reasons, some dogs missed one or more of the examinations during the study [11].

### Questionnaires and clinical registrations

History, husbandry, and clinical information for each dog were obtained from three sources: 1) the breeder of the litter; 2) the owner of the puppy; and 3) the veterinarian examining the dog. All three sources completed questionnaires and recorded information in a booklet prepared for each included dog.

The breeder recorded information regarding vaccination, deworming, nutrition, signs indicative of disease, and treatments during the first seven to eight weeks of age. Feeding regimes for each litter were decided by the breeder. All the dams had been recently vaccinated against canine parvovirus, canine distemper virus and canine adenovirus. The puppies were dewormed at two, four, and at six to eight weeks of age with fenbendazole (Panacur vet., Intervet) (some with pyranthelpamoat (Banminth vet., Pfizer)). All puppies stayed with their dam from birth to approximately seven to eight weeks of age, at which time they were sold.

The owners maintained a continuous log in a booklet regarding housing, exercise, nutrition, and health of their dogs.

The owners completed the questionnaires and reported information at three, four, six, 12, 18, and 24/25 months of age, called observational ages. Episodes of diarrhoea and vomiting were recorded in a consecutive manner in the booklet during the following observational periods: between delivery date to three months of age, three to four months of age, four to six months of age, six to 12 months of age, 12 to 18 months of age, and 18 to 24/25 months of age. Not all owners contributed with reports from all observational periods (Table 1). The booklet with questionnaires can be found online http://www.nvh.no/Documents/PDF/SportFaMed/owner_booklet2.pdf.

**Table 1:** The incidence risks of diarrhoea in the different observational periods from seven weeks to 24/25 months of age.

	Observational periods					
**Breed**	**7 weeks to** **3 months**	**3 to 4 months**	**4 to 6 months**	**6 to 12 months**	**12 to18 months**	**18 to 25 months**

LEO	35/209	14/194	28/181	14/153	11/131	7/110
	**16.7%**	**7.2%**	**15.5%**	**9.2%**	**8.4%**	**6.4%**
	(12.3 - 22.4)	(4.3 - 11.7)	(10.9 - 21.4)	(5.5 - 14.8)	(4.8 - 14.4)	(3.1 - 12.6)

NF	17/137	6/129	9/123	4/100	6/85	0/60
	**12.4%**	**4.7%**	**7.3%**	**4.0%**	**7.1%**	**0%**
	(7.9 - 19)	(2.1 - 9.8)	(3.9 - 13.3)	(1.6 - 9.8)	(3.3 - 14.6)	(0.0-6.0)

LR	24/148	10/144	16/140	9/122	7/87	6/90
	**16.2%**	**6.9%**	**11.4%**	**7.4%**	**8.0%**	**6.7%**
	(11.1 - 23.0)	(3.8 - 12.3)	(7.2 - 17.8)	(3.9 - 13.4)	(4.0 - 15.7)	(3.1 - 13.8)

IW	16/81	9/79	8/70	5/55	3/45	3/34
	**19.8%**	**11.4%**	**11.4%**	**9.1%**	**6.7%**	**8.8%**
	(12.5 - 29.7)	(6.1 - 20.3)	(5.9 - 21.0)	(3.9 - 19.6)	(2.3 - 17.9)	(3.0 - 23.0)

Total	92/575	39/546	61/514	32/430	27/348	16/294
	**16.0%**	**7.1%**	**11.9%**	**7.4%**	**5.4%**	**5.4%**
	(13.2 - 19.2)	(5.3 - 9.6)	(9.4 - 15.0)	(5.3 - 10.3)	(5.4 - 11.1)	(3.4 -8.7)

The study period is divided into six different observational periods, according to the given observational ages. Incidence risks are reported as percentages with 95% confidence intervals in brackets, with the number of episodes of diarrhoea in the numerator and the total number of reports retrieved at the observational ages as denominator. Leonberger (LEO), Newfoundland (NF), Labrador retriever (LR), and Irish wolfhound (IW).

A veterinarian examined the dog at the observational ages and recorded clinical data. Blood samples for subsequent analyses and skeletal radiographs were also taken. The dogs were vaccinated against canine parvovirus at eight weeks, three, four, 12 and 24 months, canine parainfluenza virus at three, four, 12 and 24 months and against canine distemper virus and canine adenovirus at three and 12 months of age [11].

### Descriptive statistics

Incidence risks are reported as percentages with 95% confidence intervals (the number of episodes of diarrhoea/vomiting divided by the total number of reports in a certain observational period). As all variables were skewed, distributions are presented by their median values and ranges. Episodes of diarrhoea and/or vomiting occurring less than seven days apart were calculated as one episode. Severity and duration of diarrhoea/vomiting were not taken in to consideration.

The incidence risk is calculated for each observational period separately, based on information collected at each observational age.

Four seasons (reflecting outside temperatures in Norway) were defined to account for any seasonal variation in the occurrence of diarrhoea and vomiting: winter (December to February), spring (March to May), summer (June to August) and autumn (September to November). The exact date was recorded for each episode of diarrhoea/vomiting, and the episode categorised accordingly.

Living location was categorised as urban, suburban, and rural.

### Risk factor analyses

Associations with potential risk factors for diarrhoea and vomiting were investigated in separate models. The dependent variable was dichotomous, according to whether the dogs expressed signs of diarrhoea (yes/no) or vomiting (yes/no) in each observational period. Due to repeated measurements of the dependent variables, a generalized estimating equation (GEE) using the software package Stata 11 (Stata Corporation, 4905 Lakeway Drive, College Station, TX 77845, USA) was applied. To account for correlations between observations on any given dog, GEE analyses with an unstructured correlation structure were used [13].

When diarrhoea was the dependent variable, associations with the potential risk factors: breed, gender, observational period (age), vomiting, diarrhoea in the puppy-period (birth to seven to eight weeks of age), and living location were evaluated. When vomiting was the dependent variable, associations with the potential risk factors: breed, gender, observational period (age), diarrhoea, vomiting in the puppy-period, and living location were evaluated.

The models were constructed using manual backward elimination. Predictor variables were retained in the model when the *P*-value was < 0.05. Potential intervening and confounding variables were considered after initially constructing a causal diagram. Changes of more than 20% in the coefficients in the model with the potential confounder present were also used as an indication of confounding. A variable was considered to be intervening if adding it substantially altered the effect of another variable and if the intervening variable lay on the causal path between the variable and the outcome. Biologically plausible interactions between significant predictors were tested by adding an interaction term to the final model and the interaction term was retained if *P *< 0.01. Following manual backward elimination, the model was built again by forward selection by offering the excluded variables one at a time. The multiple Wald and the likelihood ratio test were used to evaluate differences between categories of categorical predictors.

## Results

Initially 700 dogs belonging to 79 breeders were included. A total of 585 dogs (283 males and 302 females) from 568 owners participated in the study. Hence, the breeder-litter ratio in the study was 0.75 (79/106). The number of reports retrieved by the investigators from the different observational periods is listed in Table 1.

### Diarrhoea

#### Descriptive statistics

The incidence risk of diarrhoea in the four breeds in different observational periods is presented in Table 1. In the period from seven weeks to 25 months of age, 267 episodes of diarrhoea occurred in 206 dogs. Three dogs had episodes occurring less than seven days apart. Apart from these three episodes the other episodes appeared at ≥14 days apart. A total of 50 dogs had more than one episode; 41 dogs had two episodes (16 LEO, five NF, 12 LR and eight IW), and seven dogs had three episodes (three LEO, two NF, and two LR). Two dogs, all LEO, had four episodes of diarrhoea. The median time period between the start of two episodes of diarrhoea in the same dog was 152 days, ranging from 14 to 617 days.

The distribution of diarrhoea was skewed, with a median value of 128 days of age and a range of 50 to 766 days of age. The median age at the occurrence of the first and second episode of diarrhoea was 109 days (range: 50 to 726) and 184 days (range: 57 to 727), respectively.

**Figure 1:**
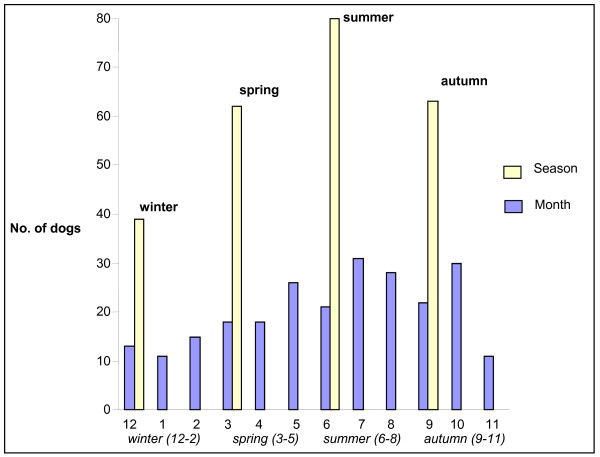
**Monthly and seasonal distributions of diarrhoea**. The year is divided into four seasons: winter (December, January and February), spring (March, April and May), summer (June, July and August) and autumn (September, October and November) reflecting outside temperatures in Norway. On the x-axis January is denoted as 1, February 2, March 3 *et cetera*.

Monthly and seasonal variations in the occurrence of diarrhoea are presented in Figure 1. The ages of the dogs with diarrhoea were not significantly different throughout the year, with young dogs comprising approximately the same proportion of the dogs in all seasons (data not shown).

#### Risk factor analyses

The incidence risk of diarrhoea was significantly affected by breed, gender, observational period, vomiting and living location (Table 2). Of the included breeds, the IW had the highest odds (OR = 1.99) of diarrhoea compared to the NF (baseline). Diarrhoea was more common in males compared to females, with OR = 1.42. After three months of age, the odds of diarrhoea were significantly lower when compared to the observational period seven weeks to three months (OR ranging from 0.31 to 0.70 depending on the period). The occurrence of diarrhoea was more common in dogs that also experienced episode(s) of vomiting during the study period (OR = 5.43). Dogs in urban areas had higher odds (OR = 1.88) of diarrhoea compared to dogs in suburban and rural areas (Table 2).

**Table 2:** Presentation of significant odds ratios for diarrhoea.

Diarrhoea		Odds Ratio	P-value	95% Conf. Interval
Living location^1^	Suburban^1^	1.31	0.06	0.99 - 1.74
	
	Urban ^1^	1.88	0.01	1.17 - 2.99

Breed^3^	LEO	1.61	0.02	1.06 - 2.43
	
	LR	1.32	0.21	0.85 - 2.02
	
	IW	1.99	0.01	1.26 - 3.15

Gender^2^		1.42	0.01	1.09 - 1.85

Vomiting		5.43	< 0.001	3.69 - 7.99

Observational period^4^	3 to 4 months	0.39	< 0.001	0.26 - 0.59
	
	4 to 6 months	0.70	0.06	0.49 - 1.01
	
	6 to 12 months	0.41	< 0.001	0.26 -0.64
	
	12 to 18 months	0.45	0.001	0.28 - 0.70
	
	18 to 24/25 months	0.31	< 0.001	0.18 - 0.55

Generalized estimating equation (GEE) analyses with an unstructured correlation structure were used. N = 585. Leonberger (LEO), Labrador retriever (LR), and Irish wolfhound (IW).

^1 ^Rural is baseline

^2 ^Male is baseline

^3 ^Newfoundland is baseline

^4 ^The observational period 7 weeks to 3 months of age is baseline

The interaction terms observational period *breed, observational period *gender, breed*living location were tested. None of the tested interactions were significant, and none of the variables were left as confounders.

### Vomiting

#### Descriptive statistics

The incidence risk of vomiting in the four breeds in different observational periods is presented in Table 3. In the period from seven weeks to 25 months of age, 164 episodes of vomiting occurred in 128 dogs. All episodes reported occurred more than seven days apart. A total of 25 dogs had more than one episode, 15 dogs had two episodes (seven LEO, six LR and two IW), and nine dogs had three episodes (six LEO, two NF, and one IW). One LR had four episodes of vomiting. The median time period between the start of two episodes of vomiting in the same dog was 96 days, ranging from 10 to 602 days.

**Table 3:** The incidence risks of vomiting in the different observational periods from seven weeks to 24/25 months of age.

	Observational periods					
**Breed**	**7 weeks to** **3 months**	**3 to 4 months**	**4 to 6 months**	**6 to 12 months**	**12 to18 months**	**18 to 25 months**

LEO	19/209	17/194	19/181	12/153	6/131	4/110
	**9.1%**	**8.8%**	**10.5%**	**7.8%**	**4.6%**	**3.6%**
	(5.9 - 13.8)	(5.5 - 13.6)	(6.8 - 15.8)	(4.5 - 13.2)	(2.1 - 9.6)	(1.4 - 9.0)

NF	9/137	2/129	1/123	0/100	2/85	1/60
	**6.6%**	**1.6%**	**0.8%**	**0%**	**2.3%**	**1.7%**
	(3.5 -12.0)	(0.4 - 5.5)	(0.1 - 4.5)	(0.0-3.6)	(0.6 - 8.2)	(0.3 - 8.9)

LR	11/148	13/144	10/140	7/122	5/87	7/90
	**7.4%**	**9.0%**	**7.1%**	**5.7%**	**5.7%**	**7.8%**
	(4.2 - 12.8)	(5.4 - 14.8)	(3.9 - 12.6)	(2.8 - 11.4)	(2.5 - 12.8)	(3.8 - 15.2)

IW	2/81	5/79	5/70	4/55	2/45	0/34
	**2.5%**	**6.3%**	**7.1%**	**7.3%**	**4.4%**	**0%**
	(0.7 - 8.6)	(2.7 - 14.0)	(3.1 - 15.7)	(2.9 - 17.3)	(1.2 - 14.8)	(0.0-10.3)

Total	41/575	37/546	35/514	23/430	15/348	12/294
	**7.1%**	**6.8%**	**6.8%**	**5.3%**	**4.3%**	**4.1%**
	(5.3 -9.5)	(5.0 -9.2)	(4.9 -9.3)	(3.6 -7.9)	(2.6 -7.0)	(2.4 -7.0)

The study period is divided into six different observational periods, according to the given observational ages. Incidence risks are reported as percentages with 95% confidence intervals in brackets, with the number of episodes of vomiting in the numerator and the total number of reports retrieved at the observational ages as denominator. Leonberger (LEO), Newfoundland (NF), Labrador retriever (LR), and Irish wolfhound (IW).

The distribution of vomiting was skewed, with a median value of 145 days of age and a range of 51 to 750 days of age. The median age at the occurrence of the first and second episode of vomiting was 117 days (range: 56 to 750) and 270 days (range: 90 to 660), respectively.

Thirty-six episodes of concurrent diarrhoea and vomiting were observed in 35 dogs.

**Figure 2:**
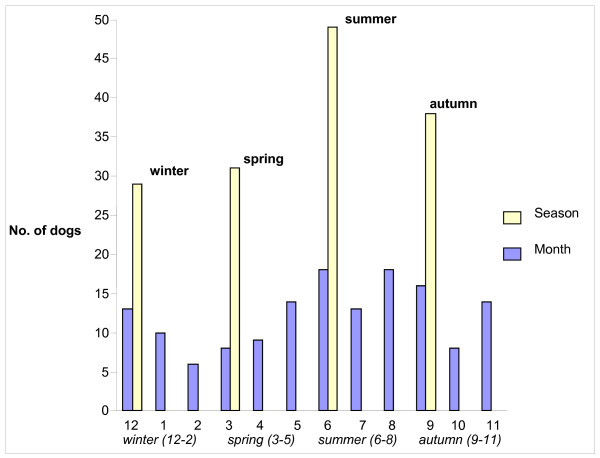
**Monthly and seasonal distributions of vomiting**. The year is divided into four seasons: winter (December, January and February), spring (March, April and May), summer (June, July and August) and autumn (September, October and November) reflecting outside temperatures in Norway. On the x-axis January is denoted as 1, February 2, March 3 *et cetera*.

Monthly and seasonal variations in the occurrence of vomiting are presented in Figure 2. The ages of the dogs with vomiting were not significantly different throughout the year, with young dogs comprising approximately the same proportion of the dogs in all seasons (data not shown).

#### Risk factor analyses

The incidence risk of vomiting was significantly affected by breed and occurrence of diarrhoea (Table 4). Of the included breeds, the LEO had the highest odds (OR = 2.93) of vomiting compared to the NF (baseline). The occurrence of vomiting was more common in dogs that also experienced episode(s) of diarrhoea during the study period (OR = 5.50) (Table 4).

**Table 4:** Presentation of significant odds ratios for vomiting.

Vomiting		Odds Ratio	P-value	95% Conf. Interval
Breed^1^	LEO	2.93	< 0.001	1.67 - 5.13
	
	LR	2.76	0.001	1.53 - 4.96
	
	IW	1.49	0.26	0.71 - 3.13

Diarrhoea		5.50	< 0.001	3.73 - 8.11

Generalized estimating equation (GEE) analyses with an unstructured correlation structure were used. N = 585. Leonberger (LEO), Labrador retriever (LR), and Irish wolfhound (IW).

^1 ^Newfoundland is baseline

The interaction terms observational period *breed, observational period *gender, breed*living location were tested. None of the tested interactions were significant, and none of the variables were left as confounders.

## Discussion

The present study reports on the incidence and risk factors of diarrhoea and vomiting in young, large breed dogs in Norway. Both diarrhoea and vomiting are relatively common conditions, although diarrhoea is more often registered. Most of the dogs only suffered from one episode of either diarrhoea and/or vomiting during the study period. Moreover, dogs suffering from several episodes of gastrointestinal disorders demonstrated relatively long periods without signs in between these episodes. In line with the findings of Hubbard *et al. *[3], a positive association between the occurrence of diarrhoea and vomiting in the same dog was found. In the majority of dogs in the present study, however, episodes of diarrhoea and vomiting did not occur at the same time.

The distributions of both diarrhoea and vomiting were skewed with a much higher frequency during the first months of life. Puppies are immunologically immature and by 12 weeks of age the majority have lost most of their maternally derived antibodies rendering them more prone to infections [14]. Additionally, the stress of weaning, transportation and re-homing could lead to an increase in gastrointestinal infections due to increased susceptibility [15]. Obviously, many other causes of acute gastrointestinal disorders, like changes in diet, ingestion of garbage or table scraps, and ingestion of foreign material are commonly reported [16]. An important issue when evaluating the health status of puppies is the owners' possible increased awareness during the first months of their dog's life. This may lead to a higher rate of registrations in the younger compared to the older dogs. Hubbard *et al. *[3] reported no association between the age of the dog and the frequency of either vomiting or diarrhoea. On the other hand, Wells and Hepper [4] reported that the frequencies of both vomiting and diarrhoea were highest in puppies and declined with increasing age. Also, studies exploring the occurrence of infectious agents, with the potential to cause gastrointestinal disorders, in faecal samples have reported a higher occurrence in young dogs [17-20].

Results from previous studies regarding a possible gender or breed influence on the occurrence of diarrhoea and vomiting in the dog, are conflicting. Hubbard *et al. *[3] did not find any association between vomiting and gender, but diarrhoea was significantly more common in males. Also, Stavisky *et al. *[21] reported a higher occurrence of diarrhoea in males in a recent case-control study. In two other studies no effects of breed and gender on the occurrence of gastrointestinal conditions were reported [4,22]. Our results indicate an association between gender and occurrence of diarrhoea, with males more often exhibiting this sign. A possible explanation may be differences in behaviour between the genders. In a study of dog to dog interaction the frequency of dog sniffing at another dog differed significantly between gender, with males more often exhibiting this behaviour [23]. Furthermore, the most common inspection areas when two dogs meet, are the head and anogenital area, with males inspecting the anogenital area more often than females [24]. Also, male dogs may show increased roaming behaviour [25]. Therefore, one could speculate whether differences in behaviour between the genders might render males at a higher risk for developing gastrointestinal disorders. In the present study, the incidence of both diarrhoea and vomiting was influenced by breed, which is in contrast to the results of Hubbard *et al. *[3]. However, some of the breeds in their study had very few individuals included. Several possible explanations for differences in occurrence of gastrointestinal conditions between breeds can be provided. Firstly, differences in genetic susceptibility leading to increased risk of infections are suggested to occur in some breeds [26]. Secondly, differences in both husbandry and behaviour might occur between different breeds. Such differences could be related to feeding regimens including the type of diet, frequency of receiving titbits, frequency of scavenging, frequency and length of walks, length of time off leash, number and length of dog to dog interactions *et cetera*. Hubbard *et al. *[3] reported differences in the frequency of scavenging between different breeds, but not the frequency of receiving titbits. Feeding a home-cooked diet, a recent history of scavenging, or change of diet all increased the risk of diarrhoea in the dog [21]. In the present study, the information about nutrition was not sufficient to assess the influence of feeding on diarrhoea and vomiting in the study population. However, almost all dogs were fed commercial diets from well-reputed international companies.

In the present study the higher incidence of diarrhoea in the urban areas could be explained by rural living locations offering fewer possibilities for dog to dog contact, and contact with other dogs' excreta. Also, in the dog, regular contact with cattle, sheep or horse faeces, which is more likely to occur in rural areas, appeared to be associated with a reduced risk of diarrhoea [21].

In the present study, the occurrence of both diarrhoea and vomiting demonstrated a seasonal variation with higher incidence in the summer months. Different seasons have the potential to influence, among other things, the frequency and length of walks, dog to dog interactions and the frequency of scavenging. Also, the occurrence of potential infectious agents responsible for diarrhoea and vomiting can be influenced by climatic conditions.

Some potential limitations of the present study need to be highlighted. A random effect for breeder was considered, but since the breeder:litter ratio was close to one, the breeder level was omitted. The GEE method is considered a suitable and robust method for analysing repeated measures with a dichotomous outcome [13], but is limited to a single level of clustering [27]. The possible effect of clustering of the dogs in litters on the two models was evaluated by using the "xtmelogit" command in Stata, with dog and litter as random effects using the same independent variables as in the GEE models (data not shown). The results from the "xtmelogit" analyses supported the GEE results, and estimates were not influenced much by the model. For diarrhoea the litter represented approximately 20% of the residual variance and the dog the rest, while the random effect was of marginal effect. For vomiting there was a strong random effect on dog, with litter explaining only 3% of residual variance. These results suggest that the GEE estimates were reliable and that the random effect at dog level was the most important one in the models. Also, episodes of diarrhoea and vomiting are owner-reported, and misclassification bias may have occurred. Diarrhoea is often defined as "increased faecal fluidity, and is usually accompanied by an increased defecation frequency and volume of faeces" [16]. This is a sign generally recognized by the owner without any problem. Vomiting is "a reflex act initiated by stimulation of the vomiting centre in the medulla" oblongata, and should be differentiated from regurgitation; "the passive evacuation of undigested food from the oesophagus" [28,29]. Vomiting is also a sign easily identified by the owner. However, regurgitation might have been classified as vomiting in our study. On the other hand, regurgitation is not so common, and usually a more permanent clinical condition compared to episodes of vomiting [3]. It should, however, be noted that some breeds, including the Irish wolfhounds, are predisposed to megaoesophagus and portosystemic shunts (PSS). These conditions, might lead to regurgitation, intermittent diarrhoea and/or vomiting [28,29]. Fortunately, the Irish wolfhounds included in the present study were all screened for PSS by a bile acid test at six to eight weeks of age and were all found to be normal (data not shown). As part of the study, all dogs were regularly assessed by a veterinarian, and none of the dogs included were diagnosed with megaoesophagus. Also, unmeasured factors, like recent stay in kennel, could confound or intervene [21].

The relatively high number of owners which left the study during the observation period could have influenced the validity. Also, the dogs in this study population were owned by people participating in a research study and they underwent regular veterinary examinations as well as regular vaccination and deworming, i.e. living under presumably optimal conditions. Stavisky *et al. *[21] reported that having up to date vaccination history reduced the risk of canine diarrhoea. Validity for other breeds (e.g. smaller breeds), other ages (e.g. older dogs), other geographical areas and dogs held under less optimal conditions can therefore be questioned. On the other hand, cohort studies generally have a high relevance to real-world situations and a relatively high external validity [30].

## Conclusion

Both diarrhoea and vomiting were relatively common in young dogs, occurring most frequently the first months of life. The incidence of diarrhoea and vomiting was significantly different between the breeds. Diarrhoea occurred more frequently in males and in dogs living in the urban areas. Also, a positive association between the occurrence of diarrhoea and vomiting in the same dog was found. In the majority of dogs in the present study, however, episodes of diarrhoea and vomiting did not occur at the same time. The occurrence of both diarrhoea and vomiting demonstrated a seasonal variation with a higher incidence during the summer months.

## Competing interests

The authors declare that they have no competing interests. Parts of the results were presented at the 20^th ^Congress of the European College of Veterinary Internal Medicine Companion Animals (ECVIM-CA) in Toulouse, France in 2010.

## Authors' contributions

BKS, CT and EMS participated in the design of the study. CT performed the statistical analyses, and all authors contributed to interpretation of the data. All authors contributed to drafting and revising of the manuscript. All authors read and approved the final manuscript.
